# Molecular cloning and characterisation of *SlAGO* family in tomato

**DOI:** 10.1186/1471-2229-13-126

**Published:** 2013-09-08

**Authors:** Zhiqiang Xian, Yingwu Yang, Wei Huang, Ning Tang, Xinyu Wang, Zhengguo Li

**Affiliations:** 1 Genetic Engineering Research Center, School of Life Sciences, Chongqing University, Chongqing 400044, People’s Republic of China

**Keywords:** AGO, miRNA, RISC, Subcellular localization, Virus defense

## Abstract

**Background:**

AGO (Argonaute) protein participates in plant developmental processes and virus defense as a core element of transcriptional regulator or/and post-transcriptional regulator in RNA induced silencing complex (RISC), which is guided by small RNAs to repress target genes expression. Previously, it was revealed that 15 putative *AGO* genes in tomato genome.

**Results:**

In present study, out of 15 detected *SlAGO* genes, only *SlAGO4C* and *SlAGO15* couldn’t be detected in roots, stems, leaves, buds, flowers and fruit of tomato by 30 cycles of PCR. *SlAGO7* could be detected in early stage of fruit (-2 dpa, 0 dpa and 4 dpa), but it was significantly down-regulated in fruit collected on the 6 days post anthesis. Moreover, *SlAGO5* could only be detected in reproductive tissues and *SlAGO4D* was specifically detected in fruit. According to blast result with miRNA database, three *SlAGO* genes harbored complementary sequences to miR168 (*SlAGO1A* and *SlAGO1B*) or miR403 (*SlAGO2A*). 5′ RACE (Rapid amplification of cDNA ends) mapping was used to detect the 3′ cleavage products of *SlAGO* mRNAs. In addition, subcellular localization of SlAGO proteins was detected. Our results showed that most SlAGO proteins localized to nucleus and cytoplasm. Importantly, nuclear membrane localization of AGO proteins was observed. Furthermore, mutated miR168 complementary site of *SlAGO1A* resulted in expanded localization of SlAGO1A, indicating that miR168 regulated localization of SlAGO1A.

**Conclusions:**

Our results contribute to demonstration of potential roles of these newly isolated *AGO* family in tomato developmental processes and proved the conserved relationships between *AGO* genes and miRNAs in tomato, which might play important roles in tomato development and virus defense.

## Background

Small RNAs regulate a lot of plant developmental and physiological processes including organ polarity, miRNA pathway, leaf and floral development [1-5]. In eukaryote, microRNA (miRNA), small interfering RNAs (siRNA), PIWI-interacting RNAs (piRNAs), scanRNAs and 21U-RNAs were produced, and these types of small RNAs were associated with different AGO family members including AGO, PIWI and group 3 proteins to act biological functions [6-9]. Guided by miRNAs, AGO proteins recognize target genes at complementary sites to repress gene translation by cleaving target mRNAs meanwhile binding to cap structure of mRNAs, and in some cases to repress gene transcription by RNA directed DNA methylation [10-14]. Typical AGO protein contains a variable N-terminal domain, a conserved C-terminal PAZ domain that recognizes the 3′ end of small RNAs, a MID (middle) that binds to the 5′ phosphate of small RNAs and a PIWI domain carrying an Asp-Asp-His (DDH) motif as an active site which exhibits endonuclease activity similar to that of RNaseH [6,7,15-17].

Each AGO protein performs biological functions differently by binding to small RNAs and direct transcriptional regulation or/and post-transcriptional regulation of target genes. For example, AGO4-like proteins combined with 24 nt small RNAs to methylate DNA [12,13]. AGO1-like proteins cleaved target mRNA or/and repressed coding process directed by miRNAs [18]. AGO2-like proteins fought against virus infection guided by siRNAs generated from double strand virus RNAs, which were synthesized by RNA-dependent RNA polymerase (RdRP) using viral RNA as templates [19]. AGO7^miR390^ complex directed synthesis of trans-acting 3 (TAS3) by recognizing two complementary sites of miR390 in primary transcript of TAS3. In addition, TAS3 regulated expression of *ARF2*, *ARF3* and *ARF4* post-transcriptionally [20,21]. Both AGO1 and AGO10 played roles in stem cell differentiation. AGO10 played as a locker of miR165/miR166 in shoot apical meristem (SAM) development, while miR165/miR166 cooperated with AGO1 to suppress SAM maintenance [22,23].

Subcellular localization of plant AGO protein help researchers to understand the mechanism of RNA induced gene silencing. Localization signals of AtAGO1, AtAGO2, AtAGO4 and AtAGO5 were detected in cytoplasm and nucleus [24-27]. Among detected AGO proteins, localization of AtAGO4 was clearly characterized so far. Along with 24 nt siRNAs, Pol-IVa, RNA DEPENDENT RNA POLYMERASE2 (RDR2) and Dicer-like 3 (DCL3), AGO4 co-localized to nuclear Cajal-bodies, site of small nuclear ribonucleoprotein (snRNP) complex maturation [28]. AGO4 also localized to a second class of nuclear bodies, called AB-bodies, which also contained other proteins involved in RNA-directed DNA methylation [26]. Recent research revealed binding to siRNA facilitated redistribution of AGO4 into nucleus [24]. Still, there are a lot of unrevealed questions about localization of AGO proteins.

AGO proteins play important roles in virus defence as core element of RISC. Mutations of AtAGO1 and AtAGO2 are hypersensitive to virus infection [19,29]. Interestingly, both AGO1 and AGO2 were found regulated by miRNA. MiR168 was considered as feedback regulator of AGO1, while AGO2 was a putative target gene of miR403 [5,29-32]. On the other hand, plant viral genome codes RISC repressors to combat with plant virus defense system. For example, 2b encoded by cucumber mosaic virus inhibits AGO1 cleavage activity [33]; Polerovirus F-box protein P0 degrades AGO1 [34]; P21 binds to miRNA/miRNA* and siRNA duplex to inhibit formation of active RISC^small RNA^ and P19 has ability to modulate the endogenous miR168 level to inhibit translational capacity of AGO1 mRNA, resulting in alleviation of the anti-viral function of AGO1 protein [35,36]. Because of the fact that virus repressors developed strategies to break AGO1-dependent plant defense system and putative mechanism that miR403 regulated AGO2 in the form of AGO1^miR403^ complex, Harvey proposed that AGO1 and AGO2 represented the first and the second layer to RNA-mediated defense and counter-defense in the interactions between plants and plant virus [19].

Recently, AGO proteins were found participated in regulation of reproduction. An *AGO* gene (*MEL1*) specifically detected in germ cell was essential for the progression of premeiotic mitosis and meiosis during sporogenesis in rice [37]. Mutations of *ago9* induced multiple gametic cells differentiation which led to multiple gametogenesis [38], and deficiency of *ago104* in Maize led to production of viable gametes without meiosis [39].

Tomato is the model plant for fleshy fruit development. However, a very few research about AGO protein on fruit formation and development of tomato was reported. Indeed, over-expression of P0 in tomato resulting in reduction of both SlAGO1A and SlAGO1B dramatically modified the radicalization of leaflets, petals and anthers [40]. However, the way by which small RNAs function remains unknown. In our study, examination of *AGO* genes expression pattern in fruit formation and development progress of tomato will be beneficial to understand potential roles of *AGO* genes in fruit development. Meanwhile, localization of AGO proteins demonstrated potential pathways of small RNAs function in tomato. Moreover, verification relationships between *AGO* genes and miRNAs reveal conserved regulation between AGOs and miRNAs, functions of which are still unknown in tomato.

## Results

### Genomic distribution, gene structure, isolation, and phylogenetic analysis of SlAGOs

**Figure 1:**
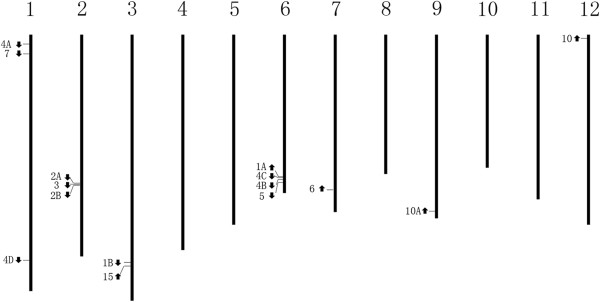
**Genomic distribution of AGO genes on tomato genome.** The arrows next to gene names show the direction of transcription. The chromosome numbers are indicated at the top of each bar.

Chromosomal locations and directions of 15 *SlAGO* genes were determined and demonstrated using BLASTN analysis on Tomato WGS Chromosomes (Figure 1, Additional file 1) [41]. *SlAGO* family genes in tomato locate in chromosome 1 (*SlAGO4A*, *SlAGO7*, *SlAGO4D*), chromosome 2 (*SlAGO2A*, *SlAGO3*, *SlAGO2B*), chromosome 3 (*SlAGO1B*, *SlAGO15*), chromosome 6 (*SlAGO1A*, *SlAGO4C*, *SlAGO4B*, *SlAGO5*), chromosome 7 (*SlAGO6*), chromosome 9 (*SlAGO10A*), chromosome 12 (*SlAGO10*), but none of the *SlAGO* genes distribute in chromosome 4, 5, 8, 10 and 11. (Figure 1 and Additional file 1). Interestingly, there was only 2096 bp in tomato genome between cloned sequences of *SlAGO3* and *SlAGO2B*, this kind of short gap was also found between genomic sequences of *AtAGO2* and *AtAGO3*. Similar protocol was used to determine exons position in *SlAGO* genes. Numbers of exons in *SlAGOs* genes ranged from 23 (*SlAGO10*) to 3 (*SlAGO2B* and *SlAGO3*), see in Additional file 1.

3′ RACE or 5′ RACE were employed to verify the predicted cDNA or EST sequences.15 predicted *SlAGO* genes were located in the tomato genome, and only 13 *SlAGO* genes CDs were cloned: *SlAGO1A*, *SlAGO1B*, *SlAGO2A*, *SlAGO2B*, *SlAGO3*, *SlAGO4A*, *SlAGO4B*, *SlAGO4D*, *SlAGO5*, *SlAGO6*, *SlAGO7*, *SlAGO10*, *SlAGO10A*. The length of sequenced cDNAs varied from 2691 bp (*SlAGO4D*) to 3729 bp (*SlAGO1B*), see results in Additional file 2. RNA used to clone *SlAGO* genes was a mix simple extracted from the whole plant of flowering tomato and fruiting tomato.

**Figure 2:**
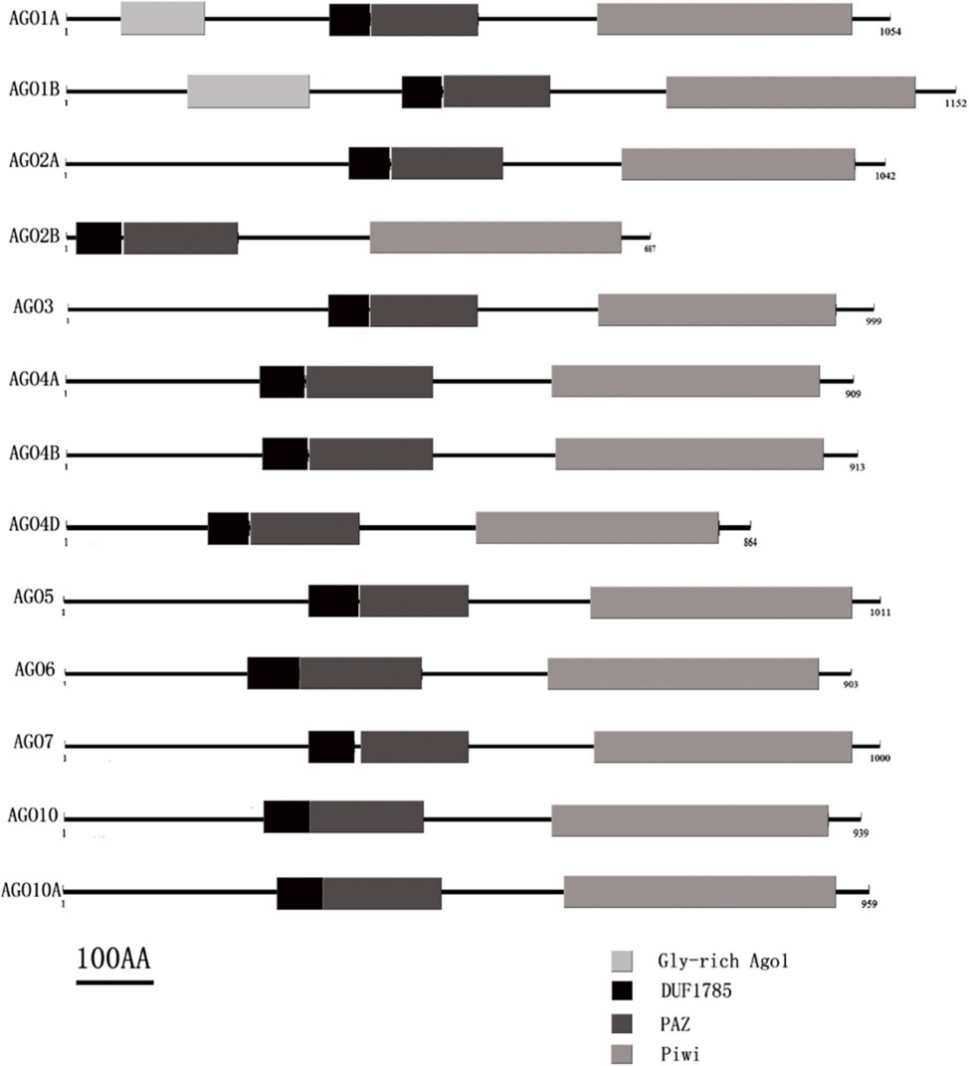
**Conserved domain in slAGOs.***Bold bars* are different conserved domain located in SlAGOs. The name of domain is above bold bar. Scale bar indicates 100 aa.

Polypeptide sequences of SlAGOs were generated by primer 5.0 in different open reading frames (ORF), the longest ones were further analyzed and verified with online tool of Pfam finder (http://pfam.sanger.ac.uk/search). The ORF length of *SlAGO* genes ranged from 2064 bp (*SlAGO2B*) to 3459 bp (*SlAGO1B*), which encoded polypeptides varied from 687 aa to 1152 aa (Additional file 3). All polypeptides of SlAGO proteins harbored DUF1785, PAZ and Piwi domain, but Gly-rich AGO1 domain was only found in ORF of *SlAGO1A* and *SlAGO1B* (Figure 2). High ratio of G and Q were found in N terminus of SlAGO1A, SlAGO1B, SlAGO2, SlAGO3, SlAGO5 polypeptides and G-Q percentage were analyzed in N terminus domain. There were15.2% G and 9.5% Q in 335 aa N terminus of SlAGO1A, 17.9% G and 14.9% Q in 436 aa N terminus of SlAGO1B, 16% G and 9.6% Q in 374 aa N terminus of SlAGO2A, 12.8% G and 6.1% Q in 312 aa N terminus of SlAGO5 (Additional file 4).

**Figure 3:**
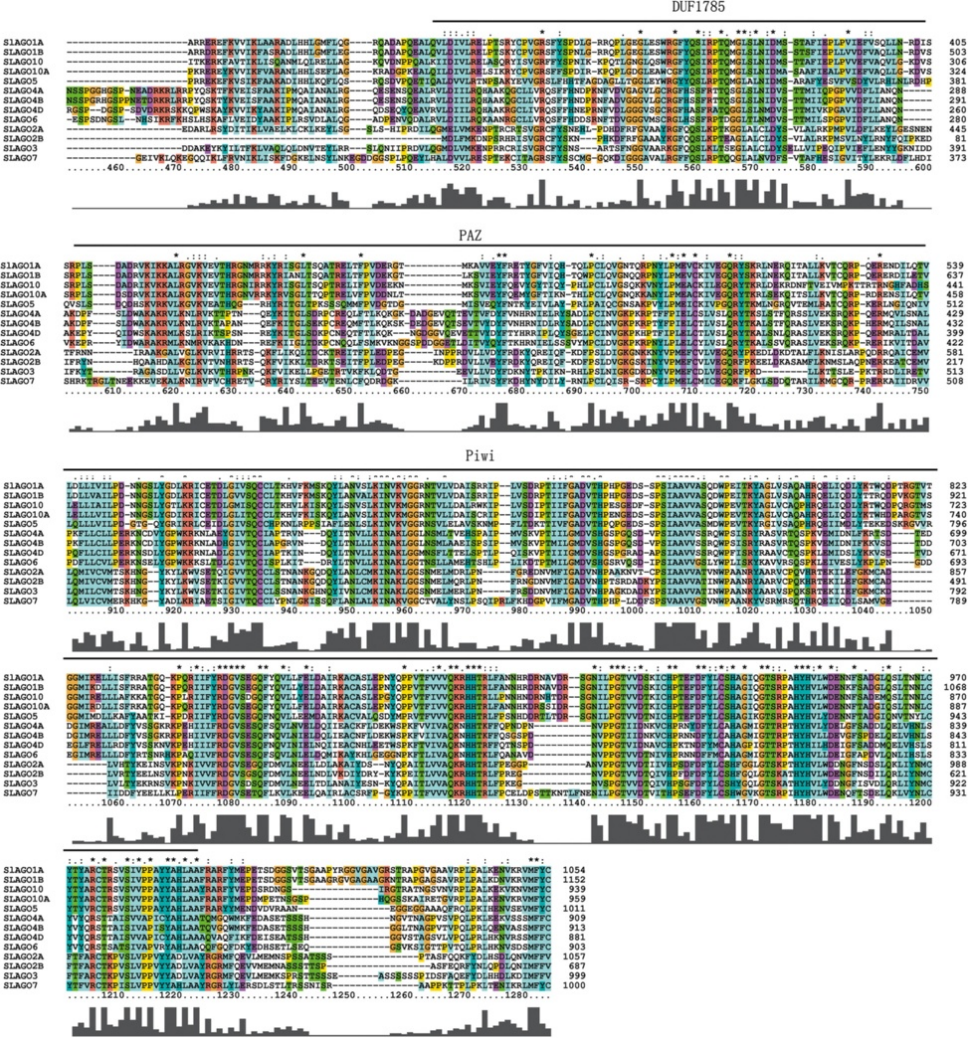
**Multiple sequence alignment of the full-length SlAGO proteins obtained by ClustalX and manual correction.** Conserved domains of AGO proteins are underlined.

Interestingly, although 2995 bp of *SlAGO2B* cDNA was amplified in our work, only 2064 bp which coded DUF1785, PAZ and Piwi domains were found in the ORF with 7 stop signals presented in 5′ terminus sequence (Figure 3). Three individual clones of *SlAGO10A* were sequenced and 3160 bp fragment of *SlAGO10A* was amplified, but a stop signal presented at the position of 594 aa in the predicted ORF coding 997 aa. Alignment were performed between predicted cDNA and sequenced cDNA. An extra sequence “TAGTTGTTTTGCAACCTCTTTCTCTTTTTTTCCTTGTCCATTTCTCTTTCAGGTCGATGTTTGTTAAAGACACGAAGACGATTGACCTTTGCTCCTGTGCGTTATGCTGACAGG” was found in sequenced cDNA, which located in chromosome 9 from 63891277 to 63891165, the 11th exon of *SlAGO10A* genomic sequence (ch9: 63895090–63888840). But the stop signal was alternatively spliced in EST sequence [SGN:SGN-U604148] which was 100% matched to predicted cDNA of *SlAGO10A*. A *SlAGO10A2* [NCBI: JX467717] alternative splicing cDNA was generated according to our results and SGN-U604148. 5′ RACE results of *SlAGO2A* and one clone of *SlAGO2A* CDs showed an extra “GCCGGCAGAGGTGGAGGTAACC” localized to SL2.40ch02: 33782189–33782210 and an extra “TTTTGCTGTGAACTGGTCTGACGG” localized to SL2.40ch02: 33785522–33785545, which coded “AGRGGGN” (22 aa to 28 aa) and “FAVNWSDG” (314 aa to 322 aa) respectively. However, these two fragments were not detected in another clone of *SlAGO2A2* [NCBI: JX493012], while both of them were located in N terminal variable domain.

**Figure 4:**
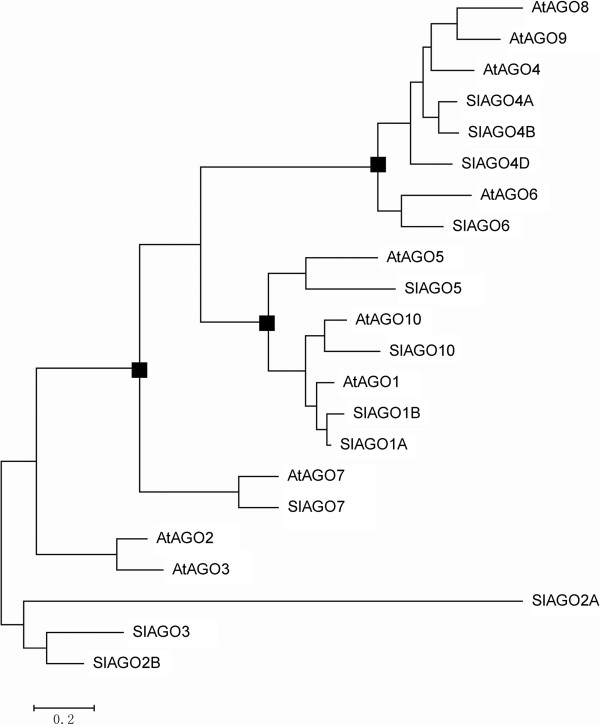
**Phylogenetic relationships between tomato and *Arabidopsis *AGO proteins.** The phylogenetical tree was generated using MEGA5.0 program by the Maximum Likelihood method. Sequences access numbers were listed in Additional file 2 and Additional file 10.

The phylogenetic tree was generated from alignment of full-length protein sequences of the 12 cloned *SlAGO* genes (*SlAGO10A* couldn’t generate complement proteins) and the 10 *AtAGO* genes [29]. Phylogenetic analysis showed that 12 SlAGO proteins divided into three major clades which were similar to those in *Arabidopsis* (AtAGO1-like, AtAGO4-like and AtAGO2-like class, see Figure 4). AtAGO1-like clade included SlAGO1A, SlAGO1B, SlAGO5, SlAGO10; AtAGO4-like clade included SlAGO4A, SlAGO4B, SlAGO4D, SlAGO6; AtAGO2-like clade included SlAGO2A, SlAGO2B, SlAGO3 and SlAGO7 (Figure 4). Interestingly, SlAGO proteins which were grouped into the clade of AtAGO1 or AtAGO4 possessed at least 20 exons, while SlAGO proteins which were grouped into the clade of AtAGO2 only had 5 to 3 exons (Additional file 1). Sequence information, genomic distribution, gene structure and multiple alignments of polypeptide were presented in Additional file 2, Figures 1, 2 and 3 respectively.

#### ***SlAGO1A, SlAGO1B and SlAGO2A are target genes of conserved miRNA***

**Figure 5:**
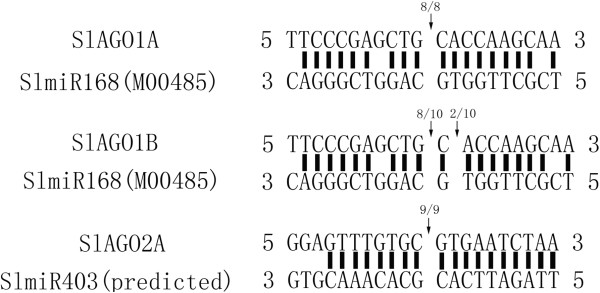
**Cleaved site of miRNA at complimentary site of *AGO *genes.***SlAGO1A* was cleaved between 10 and 11 nt of complimentary sequence to miR168; *SlAGO1B* was cleaved between 10 and 11 nt of complimentary sequence to miR168; *SlAGO2B* was cleaved between 10 and 11 nt of complimentary sequence to miR403.

13 cloned *SlAGOs* cDNA sequences and 2 predicted cDNA of *SlAGO4C*, *SlAGO15* were performed blast in tomato putative miRNA database and conserved plant miRNA in miRBase [42-44]. *SlAGO1A* and *SlAGO1B* were found complementary to mature SlmiR168 [TFGD: M00485], and *SlAGO2A* was found complementary to mature AtmiR403. Putative pre-SlmiR403 was found in tomato genome and its structure was folded by RNA Folder version 1.11 (Additional file 5 and Additional file 6). Cleavage sites of complementary sequence to miRNAs were detected by 5′ RACE. 8 over 8th positive clone showed cleavage site localized between 10th and 11th nt of miR168; 8 over 10th positive clone showed cleavage site localized between 10th and 11th nt of miR168; 9 over 9th positive clone showed cleavage site localized between 10th and 11th nt of miR403 (Figure 5).

### SlAGOs proteins localized to cytoplasm and nucleus

**Figure 6:**
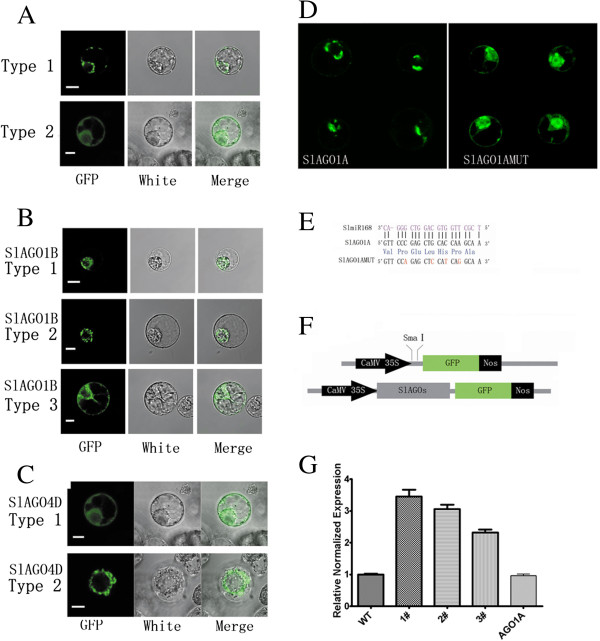
**Subcellular localization of SlAGO proteins in BY2 protoplasts.** Plasmid harboring CaMV 35S:SlAGOs-GFP tag were transformed into BY2 cell. **(A)** Two types of localization of SlAGO proteins. Type 1 localized to membrane of cytoplasm and nucleus; Type 2 localized to cytoplasm and nucleus. More localization of SlAGO proteins were listed in Additional file 7. **(B)** Different subcellular localization of AGO1B in different cells. Type 1 localized to nucleus; Type 2 localized to membrane of nucleus; Type 3 localized to cytoplasm and nucleus. **(C)** Different subcellular localization of AGO4D in different cells. Type 1 localized to cytoplasm and nucleus; Type 2 localized to ring structure in nucleus. **(D)** Subcellular localization of AGO1A and AGO1AMUT. **(E)** Site mutation strategy of SlAGO1A at complimentary sequences to mature SlmiR168. Red letters in AGO1AMUT sequence stand for mutation sites. Blue letters were amino acids coded in the open read frame. **(F)** Strategy for recombination of SlAGOs:GFP. The scale indicates 5 μm. **(G)** Resistance test of *SlAGO1AMUT* in transgenic plant. 1#, 2#, 3# were individual transgenic plants of *SlAGO1AMUT*; SlAGO1A was transgenic plant of *SlAGO1A*; WT was wild type micro-TOM.

CaMV:SlAGOs-GFP vectors were constructed and transformed into BY2 protoplast and subcellular localization of SlAGOs were observed. Generally, there were two types of subcellular localization of SlAGOs: Type 1 which includes SlAGO1A, SlAGO5 and SlAGO10 localized to nuclear membrane and cell membrane, while type 2 including SlAGO2A, SlAGO3, SlAGO4A, SlAGO4B and SlAGO6 expanded to cytoplasm and nucleus (Figure 6A, Additional files 7, 8 and 9). Interestingly, SlAGO1B localized to nucleus (Figure 6B, Type 1), to cytoplasm (Figure 6B, Type 2) or both to cytoplasm and nucleus (Figure 6B, Type 3) and SlAGO4D localized to cyt oplasm and nucleus (Figure 6C, Type 1) or localized around nucleus (Figure 6C, Type 2). SlAGO1A localized to spot structure around nucleus (Figure 6D). Moreover, subcellular localization of mutation at the complementary site to miR168 without altering polypeptide sequence of SlAGO1A expanded to nucleus and cytoplasm, not only spot structure around nucleus as the subcellular localization of SlAGO1A (Figure 6D, Figure 6E).

### Different expression pattern of *SlAGO* genes

**Figure 7:**
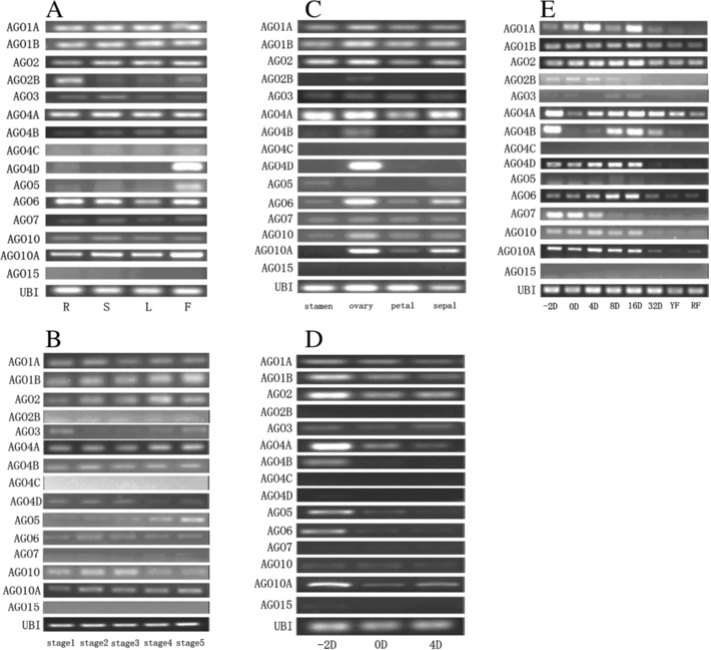
**PCR analysis of *SlAGO *genes in tomato. (A)** Expression patterns of *SlAGO* genes in different organs. R, S, L, F stands for roots, stems, leaves and flowers collected from flowering tomato. **(B)** Expression patterns of *SlAGO* genes during bud development. Buds were divided in to 5 developmental stages from stage 1 to stage 5 according to the length of bud. Stage 1 stands for no more than 1 mm; Stage 2 stands for between 2 to 3 mm, stage 3 stands for between 4 to 5 mm, stage 4 stand for between 6 to 7 mm and stage 5 stands for between 8 to 9 mm. **(C)** Expression patterns of *AGO* genes in different organs of tomato flowers. Stamens, pistil, petals and sepals were collected from flowering plants. **(D)** Expression patterns of tomato *AGO* genes during stamen development. -2D, 0D and 4D stands for stamens collected from flowers 2 days before anthesis, the very day of anthesis and 4 days post anthesis respectively. **(E)** Expression patterns of *SlAGO* genes during tomato fruit development. -2D stands for fruits collected 2 days before anthesis; 0D, 4D, 8D 16D and 32D stands for fruits collected on the 0, 4, 8, 16 and 32 day post anthesis respectively; YF stands for yellow fruits and RF stands for red fruits. *Ubi3* was used as a reference gene to normalize expression level and 30 cycles of PCR were performed for each primer.

Previously, expression patterns of *SlAGOs* were detected in shoots, roots, flowers, mixture of green fruit and red fruit, and *SlAGO4C, SlAGO4D* and *SlAGO15* could not be detected [45]. In our study, *SlAGO4C* and *SlAGO15* could not be detected in any of collected tissues as well, but full CDs of *SlAGO4D* were cloned and *SlAGO4D* could be detected in flower and early stages of fruit development. Expression patterns of *SlAGO* genes were detected in roots, stems, leaves and flowers. As a result, *SlAGO1A, SlAGO1B, SlAGO2A, SlAGO2B, SlAGO3, SlAGO4A, SlAGO4B, SlAGO6, SlAGO7, SlAGO10* and *SlAGO10A* could be detected in all four organs, while *SlAGO4D* and *SlAGO5* could be detected in flowers only (Figure 7A).

According to length, flower buds were grouped into 5 stages: stage 1 was flower buds when the length was no more than 1 mm, stage 2 was 2 to 3 mm, stage 3 was 4 to 5 mm, stage 4 was 6 to 7 mm and stage 5 was 8 to 9 mm flower buds. *SlAGO4C* and *SlAGO15* could not be detected in any collected stages and *SlAGO5* displayed gradually enhanced expression during bud development, *SlAGO7* could be detected on stage 4 and stage 5 of flower buds only (Figure 7B).

Flowers were divided into stamens, ovaries, petals and sepals. *SlAGO1A, SlAGO1B, SlAGO2, SlAGO3, SlAGO4A, SlAGO4B, SlAGO6, SlAGO7, SlAGO10* and *SlAGO10A* could be detected in all four parts, but expressions of *SlAGO6* and *SlAGO10A* in ovaries were significantly higher than in other parts of flowers. *SlAGO2B* and *SlAGO4D* could be detected in ovaries but not in other parts of flowers (Figure 7C).

Stamens were collected on 2 days before anthesis (-2 dpa), the first day of anthesis (0 dpa) and 4 days post anthesis (4 dpa) respectively. *SlAGO2B, SlAGO4C, SlAGO4D, SlAGO15* could not be detected in any of these collected stages when 30 cycles of PCR was performed. *SlAGO1A, SlAGO1B, SlAGO2, SlAGO3, SlAGO4A, SlAGO5, SlAGO6, SlAGO10* and *SlAGO10A* could be detected in three stamen developmental stages, while *SlAGO4B* could be detected in -2 dpa stamens (Figure 7D).

To understand function of *SlAGO* genes in fruit development, fruit were harvested on 2 days before anthesis (-2 dpa), the first day of anthesis (0 dpa), 4 days post anthesis (4 dpa), 8 days post anthesis (8 dpa), 16 days post anthesis (16 dpa), 32 days post anthesis (32 dpa), about 45 days post anthesis (yellow fruit, YF) and about 50 days post anthesis (red fruit, RF). *SlAGO1A, SlAGO1B, SlAGO2A, SlAGO2B, SlAGO4A, SlAGO4D, SlAGO6, SlAGO10* and *SlAGO10A* could be detected in all collected stages. Expression of *SlAGO4A* and *SlAGO4B* in -2 dpa fruit was significantly higher compared to that in 0 dpa fruit, but gradually up-regulated in 4 dpa fruit, reached the peak in 16 dpa fruit and followed by down-regulation in 32 dpa fruit, yellow fruit and red fruit. *SlAGO4D* and *SlAGO6* shared similar expression pattern with *SlAGO4A* and *SlAGO4B*, but *SlAGO4A* and *SlAGO4B* expression strongly in 0 dpa fruit. Slightly expressed *SlAGO5* was detected in 8 dpa fruit and 16 dpa fruit. Interesting expression pattern was found in *SlAGO7*, which expressed highly in -2 dpa fruit and slightly down regulated in 0 dpa fruit and 4 dpa fruit, but dramatically reduced in the detected stages after 4 dpa (Figure 7E).

## Discussion

In eukaryote, AGO proteins are conserved gene family and the core part in all-known small-RNA-directed regulatory pathways which regulate developmental progress by repressing expression of the target genes [13,18]. For example, AGO1 and AGO10 regulate shoots apical meristem under the direction of miR165/miR166 [22]; AGO7 affects develop timing and patterns under the direction of TAS3 [20,46]. In this study, out of 13 cloned *SlAGO* genes, 12 have generated complete protein, while *SlAGO10A* had a stop signal in predicted ORF. 12 SlAGOs and 10 AtAGO proteins were grouped into three clades by phylogenetic analysis (Figure 4) [29]. Each of *SlAGO* gene has homologous gene in *Arabidopsis*, indicating that *SlAGO* genes might have similar functions in tomato as *AtAGO* genes in *Arabidopsis*.

Homology of SlAGO1A and SlAGO1B was 88.0%, but SlAGO1A and SlAGO1B might play different roles in tomato development. There was an extra 101 aa GQ-rich sequence in the N terminus of SlAGO1B, and the percentage of Q in SlAGO1B was much higher than that in SlAGO1A (14.9%:9.5%, Additional file 4). Expression of *SlAGO1A* was different from that of *SlAGO1B* in fruit, which was much higher in 4 dpa and 16 dpa fruit compared to other detected stages of fruit development, while expression of *SlAGO1B* soldierly changed in all detected tissues (Figure 7E). SlAGO1A localized to spot around nucleus (Figure 6E), but SlAGO1B localized to nucleus (Figure 6B, Type 1), to cytoplasm (Figure 6B, Type 2) or both to cytoplasm and nucleus (Figure 6B, Type 3). Different subcellular locations and expression patterns shown two *AGO1-like* genes might function differently in tomato.

As known, miRNAs guide AGOs to regulate target genes, meanwhile *AtAGO1* was regulated by miR168 and *AtAGO2* was regulated by miR403 respectively [5,29-32]. In *Arabidopsis*, Co-IP experiment presented AGO1 bound with miR168 and miR403 [NCBI:GSE22252] and AGO2 bound with miR168 as well [5,22,47], which demonstrated multiple AGO proteins participated in post-transcript regulation of AGO1 protein via miR168 and *AGO2* mRNA might be regulated by miR403 in the form of AGO1^miR403^ complex. Moreover, miR168 and miR403 are conserved microRNAs, which can be detected in tomato (http://bioinformatics.cau.edu.cn/cgi-bin/PMRD/expression/probe_detail_3.cgi?page=1). In our study, *SlAGO1A* and *SlAGO1B* were found to be cleaved by miR168, and *SlAGO2A* was found to be cleaved by miR403. Our findings proved relationships between miR168 and *AGO1*, miR403 and *AGO2* were conserved in tomato. Unlike the feedback control of miR168 to *AGO1* is important for proper plant development, importance of miR403-mediated *AGO2* is still unknown [5,30,31]. However, recent researches might reveal the mystery of the relationship between miR403 and *AGO2*. As miR168 and miR403 were down-regulated when plants receive virus infection signal and both *ago1* and *ago2* mutations were hypersensitive to virus infection, AGO1 and AGO2 represented the first and second layer to RNA-mediated defense and counter-defense in the interaction between plants and plant-virus [19,29,48,49]. Based on these facts, a hypothesis of relationship between miR403, miR168 and biological function of AGO1, AGO2 in virus defense was presented:

As the key element in RISC, AGO proteins was up-regulated during virus infection, meanwhile over-expression of AGO proteins would induce plant development disorder [5,45]. So regulation of AGO proteins to maintain suitable amount in both condition are crucial for virus defense and plant development. Virus employ strategies to interfere RNA induced gene silencing depending on AGO proteins. There are four strategies of viral suppression RNA induced silencing process which had been identified to date: 2b interferes activate site of AGO protein; P19 and P21 inhibit formation of RISC^vsiRNA^ formation, moreover P19 is found to induce miR168 accumulation which represses expression of AGO1; the polar virus F-box protein P0 can bind to AGO1 then degrade AGO1 to suppress RNA induced silencing of viral genes [33-35,50]. These strategies show the importance of AGO proteins in RNA induced silencing-depended defense system, and no matter what strategies virus employ, up-regulation of AGO proteins in plant is crucial for success in defense against virus.

During viral infection, miR168 was found down-regulated at the early stage and up-regulated at the later stage [48]. Expression of miR168 indicates the following regulation pathway: At the beginning of virus infection, miR168 is down-regulated, AGOs^miR168^ complex are reduced subsequently. As losing post-transcriptional repressor, accumulation of AGO1 protein is initiated, and defense of virus infection is performed mainly by AGO1^vsiRNA^. At later stage, miR168 is up-regulated, AGO1^miR168^ and AGO2^miR168^ act as feed-back regulation to maintain suitable AGO1 proteins for plant development. But when AGO1 is suppressed, accumulation of AGO1^miR403^ is lower than that under ordinary condition, consequently miR403 is down-regulated during virus infection as AGO can protect binding miRNAs [30,49]. Then AGO2 is accumulated as a result of reduced repression from AGO1^miR403^, so viral RNAs are wiped out by AGO2^vsiRNAs^.

Recently, *AtAGO9* and *ZmAGO104*, which were homologous to *AtAGO4*, were found to have the ability to repress somatic fate in germ cells [38,39]. In present work, 13 *AGO* genes were cloned and expression patterns in different organs and developmental stages in stamens and friut were detected. Reversal “V” style expression patterns of *SlAGO4A, SlAGO4B, SlAGO4D, SlAGO6* in fruit development were found. Interestingly, these *SlAGO* genes were grouped into *AtAGO4* clade which might play similar roles in tomato fruit development as well as *AtAGO9* and *ZmAGO104* did, especially the candidate *SlAGO4D* which specifically expressed in friut.

In rice, an *AtAGO1-like* gene *MEL1* expressing in reproductive organs regulates cell division of premeiotic germ cells [37]. In tomato, there are 5 *SlAGO* genes belonged to *AGO1* clade (*SlAGO1A, SlAGO1B, SlAGO5, SlAGO10* and *SlAGO10A*). Among five *SlAGO1-like* genes, *SlAGO5* expresses in flowers and fruit, the expression pattern of which is similar to that of *MEL1*, indicating that *SlAGO5* might function in regulation of reproductive organ formation in tomato.

The unique expression pattern during fruit development indicated that *SlAGO7* might participate in formation of fruit. There were evidences that could demonstrate potential pathway. First, AtAGO7 was found participating in synthesis of *trans-acting 3* by direction of miR390, and *AtARF2, AtARF3, AtARF4* were regulated by TAS 3 [20,46]. Second, *AtARF3* and *AtARF4* had ability to mediate organ asymmetry. Moreover, mutant phenotype of *arf3* was restricted in flowers [51]. Third, over-expression of miR-ARF interfered with expression of *ARF2*, *ARF3* and *ARF4*, resulting in abnormal short style and thickened green stigma [52]. *SlAGO7* expressed extremely high in -2 dpa fruit and was dramatically down-regulated in fruits collected after 8 dpa to red fruit, indicating that *SlAGO7* which is homologous to *AtAGO7* might regulate early stage of fruit formation, most likely through directing synthesis of TAS 3 to maintain suitable expression of *ARF2, ARFA3* and *ARF4*.

Localization of AGO protein helps understand mechanism of small RNAs generation and their functions. AtAGO1 localized to both nucleus and cytoplasm and a large nucleoplasmic signal was observed [27]. AtAGO2, AtAGO5 and AtAGO4 were found to localize to cytoplasm and nucleus [24-26]. In our study, subcellular localizations of 10 SlAGOs were detected. SlAGO1A, SlAGO5 and SlAGO10 localized to nuclear menbrane and cell membrane (Figure 6A, Type 1), while SlAGO2, SlAGO3, SlAGO4A and SlAGO4B localized to cytoplasm and nucleus. Interestingly, unlike other AGO1-like genes, localization of SlAGO1A seemed to be restricted in specific structure around nucleus in tomato (Figure 6D), the function and mechanism of which is still unknown. Localizations of AGO1-like genes in tomato indicated the potential for AGO^miRNA/siRNA^ monitoring target mRNAs when mRNAs were transporting out of nucleus before translation initiating.

To check localization under the mimic condition of losing control of miR168, SlAGO1AMUT which harbored four points mutation at complementary site of miR168 was employed. Localization of SlAGO1AMUT expanded to nucleus and cytoplasm (Figure 6D and Figure 6E), indicating miR168 might regulate localization of SlAGO1A. In recent years, it was found that nucleus localization of AGO4 could be determined by binding to small RNAs [24]. Differently, AGO1 could bind to a double stranded RNA (DsRNA) binding domain protein Hyponastic Leaves1 (HYL1) in D-body, indicating that formation of mature miRNA and resemble of AGO1^miRNA^ complex were coupled in nucleus [27]. In our study, fluoresces of SlAGO1A: GFP and SlAGO1B: GFP were not strong in nucleus (Figure 6B and Figure 6D). But *SlAGO1AMUT* expanding localization to nucleus indicates that localization of SlAGO1A protein is adjusted by feedback regulation of miR168. As the fact that siRNAs and miRNAs are generated from cytoplasm and nucleus respectively, when miR168 is down-regulated, subsequently SlAGO1A protein is transported to cytoplasm and nucleus in order to accelerate formation of SlAGO1A^siRNA/miRNA^ complex. Then, suitable protein expression of target genes is monitored by SlAGO1A^siRNA/miRNA^ complex.

## Conclusion

In this study, 13 *SlAGO* genes were cloned and their expression patterns in fruit development were detected in tomato. Conserved relationships between SlAGO genes and miRNAs were discovered and identified in tomato. Moreover, localizations of SlAGO proteins and regulation of SlAGO1A localization by miR168 were observed. In tomato, identification and characterisation of AGO proteins will be beneficial to the research on transcriptional and post-transcriptional regulation of fruit formation and development through RISC pathway.

## Methods

### Searching for the ***AGO*** genes

There were 15 putative *AGO* genes persisted in tomato genome [45]. The 15 potential *SlAGO* genes were verified by multiple database searches. BLASTN and TBLASTN were performed to find previously identified AGO proteins using database of SGN tomato combined-WGS, BAC, and unigene sequences; BLASTN and TBLASTN were used to obtein potential *AGO* family genes in tomato from database of Tomato WGS Chromosomes (sl2.40) (http://solgenomics.net/tools/blast/index.pl) [41]. Based on the combined results from all searches, 15 potential *AGO* genes were identified from the currently available genomic database. After searching for *AGO* genes, bioinformatics tools, such as FGENESH (http://linux1.softberry.com/berry.phtml?topic=fgenesh&group=programs&subgroup=gfind) was used to analyze and predict those unknown *SlAGOs*, and BLASTX of NCBI (http://blast.ncbi.nlm.nih.gov/Blast.cgi?PROGRAM=blastx&BLAST_PROGRAMS=blastx&PAGE_TYPE=BlastSearch&SHOW_DEFAULTS=on&LINK_LOC=blasthome) was used to identify the functional domains. The predicted cDNA sequences of 15 putative *AGO* genes were blast to tomato genome and about 500 bp putative 3′ UTR and 5′ UTR were separated to blast with EST database to find data of 3′ UTR and 5′ UTR sequences (Additional file 2).

### Cloning of ***SlAGO*** genes

Primers designed according to predicted CDs or EST sequences were used to clone full CDs or performed 5′ RACE/3′ RACE products of each *AGO* gene (Additional files 10 and 11). Total RNA was extracted from tissues of whole flowering tomato and fruiting tomato using TRIZOL reagent (Invitrogen, Germany) according to the manufacturer’s instructions. After DNaseItreatment, 35 cycles of PCR was performed to amplify *Ubi3* using treatmented RNAs as templates, no fragments should be detected. The first cDNA strand was generated using RevertAid™ First Strand cDNA Synthesis Kit (Fermentas, Thermo SCIENTIFIC, USA) following the manufacture’ protocol. The full-length of 13 *SlAGOs* were amplified by PCR using primers designed based on the predicted cDNA from Slc4.0 (Additional file 1). 3′ RACE and 5′ RACE were performed with the 3′-Full RACE Core Set Ver.2.0 and 5′-Full RACE Kit (TAKARA, JAPAN) using primers designed according to sequencing result of cDNAs. Fragments were cloned to pEasy-blunt vector (Transgene, China) and 3 positive clones were sent to sequence by Genscript (China).

### Muitiple-sequence alignment and phylogenetic analysis

Gene sequences were analyzed by primer 5.0 and the ExPASY Proteomic Server (http://web.expasy.org/translate/). Multiple-sequence alignment was analyzed using the ClustalX (2.0.10) program and Phylogenetic analysis was performed using MEGA5 (UPGMA). Sequences accession numbers were listed in Additional file 3 and Additional file 12.

### Expression analysis of *SlAGO* genes

The plant materials were collected from Micro-Tom plants which were grown in green house under controlled temperature. The roots, stems, leaves and flowers were collected from flowering tomato plants. -2 dpa fruit were collected 2 days before anthesis, and 0 dpa fruit, 4 dpa fruit, 8 dpa fruit, 16 dpa fruit, 32 dpa fruit were collected on 1, 4, 8, 16 and 32 days post anthesis respectively. Yellow and red fruit were picked up respectively according to their color. Each collection were harvested from 10 plants and thoroughly mixed.

RNA extraction and reverse transcription was described as mentioned before. PCR was carried out to detect expression of each *SlAGO* gene using the primer pairs listed in Additional file 11, which were designed according to the 3′ terminal sequences of each *SlAGO* gene. A sample of 10 times diluted cDNA (1 μL) was subjected to PCR in a final volume of 25 μL containing 12.5 μL PCR master Mixture (Comwell, China) in the PCR machine (bio-RAD), programmed to heat for 4 min at 95°C, followed by 30 cycles of 30 s at 95°C, 30 s at 58°C and 30 s at 72°C. PCR was repeated three times individually. To normalize the total amount of cDNA presented in each reaction, the *Ubi3* gene was co-amplified as an endogenous control.

### Relationships between *SlAGOs* and miRNA

To identify the relationship between *SlAGO* genes and small RNAs, BLAST search of the tomato small RNA database (http://ted.bti.cornell.edu/cgi-bin/TFGD/sRNA/target.cgi) and miRBase (http://www.mirbase.org/) were performed using cDNA sequences of 13 sequenced *AGO* genes and predicted cDNA sequences of another 2 *AGO* genes. 5′ RACE mapping was taken to identify the slicing site of *AGO* mRNAs which were complementary to miRNAs. cDNA used to detect 5′ RACE mapping of 3′ cleaved products was reverse-transcripted after linking an adaptor by T4 RNA ligase (Fermentas, Thermo, USA). PCR was performed according to Tm of each GSP primers (Additional file 13), which were designed at the predicted 3′ products of complementary site of mature miRNA sequences. Adaptors and linker were bought from Takara (Takara 5 RACE KIT, Japan). PCR products were linked to pEasy-blunt vector and 8 to 9 positive cloned were sent to sequencing for each product.

### Site mutation of *AGO1A* by recombinant PCR

According to complementary site of SlmiR168 in sequence of *SlAGO1A* mRNA, four base mutated primers (AGO1AMUT-F and AGO1AMUT-R, Additional file 11) were designed to amplify mutated *AGO1AMUT*. AGO1A-F and AGOMUT-R were for short fragment amplification, AGO1AMUT-F and AGO1A-R were for long fragment amplification. Then each 1 μL purified mutated fragment was mixed to amplify full length of *AGO1AMUT*. This resistant fragment and original *SlAGO1A* were constructed to plp-100 driven by CaMV-35S promoter then transfected into tomato by *Agrobacterium*. Resistance to miR168 of *SlAGO1AMUT* was tested in three individual transgenic plants with the primer qSlAGO1A-cutting-F: GTGGAATAGCCCCTCAACAGTC and qSlAGO1A-cutting-R: TTGGTTCAGGTGGCTGAGATG, which was designed at each side of cutting site, UBI was employed as reference gene for normalization (Figure 6G).

### Subcellular localization of SlAGOs-GFP fusion protein

The coding sequences of *SlAGO* genes were cloned as a C terminal fusion in-frame with the green fluorescent protein (GFP) into the pGreen vector and expressed under the transcriptional control of the cauliflower mosaic virus 35S promoter and the nopaline synthase (nos) terminator [53]. Protoplasts of suspension-cultured tobacco (*Nicotiana tabacum*) BY-2 cells were produced and PEG transformation was carried out to observe cellular localization of SlAGO proteins according to the method described by Abel and Sheen (http://molbio.mgh.harvard.edu/sheenweb/protocols_reg.html) [54,55]. 2 g BY2 cells of 6 to 8 d were collected by centrifuging at 3500 rpm for 15 min, and washed twice with Tris-MES buffer; enzymes treatment was carried out at 37°C for 1 h in Tris-MES buffer digested with 1% caylase, 0.2% pectolyase Y-23 and 1% BSA. Protoplast was filtered through nylon and washed by W5 buffer, and re-suspended in MMg Buffer, counted under the microscope, then adjusted at final concentration of about 1 × 10^6^ mL^-1^ . 0.2 mL protoplast suspension was transfected with 50 μg shared salmon sperm carrier DNA, 30 μg of 35S:SlAGOs-GFP and 40% PEG for 1 h. Transfected protoplasts were centrifuged at 1000 rpm for 8 min followed by suspending in W5, then incubated for at least 16 h at 25°C. GFP fluorescence was analyzed by confocal microscopy. More than 4 illuminated cells for each SlAGOs-GFP transient expression were observed.

## Competing interests

The authors declare no competing interests.

## Authors’ contributions

ZQX, ZGL and YWY designed the study. ZQX collected the datasets from databases and analyzed the data, then prepared the original draft the manuscript. ZQX and WH constructed vector used in this study. ZQX, NT and XYW compliment the part of BY2 transient transformation and observation. All authors read and approved the final manuscript.

## Supplementary Material

Additional file 1 **Localization and exon-intron mapped in chromosomes of *SlAGO *genes.** Click here for file

Additional file 2 **Basic informations of 13 cloned *SlAGO *genes.** Click here for file

Additonal file 3 Information of SlAGO proteins and NCBI access numbers. Click here for file

Additional file 4 G-Q content in N terminal sequences before domain of DUF1785. Click here for file

Additional file 5 Information of putative SlmiR403. Click here for file

Additional file 6 Stem-loop structure of pre-SlmiR403. Click here for file

Additional file 7 Subcellular localization of SlAGO proteinss. Click here for file

Additional file 8 Type 1 localization of SlAGOs which localized to membrane of nucleus and cell. Click here for file

Additional file 9 Type 2 localization of SlAGOs which localized to nucleus and cytoplasm. Click here for file

Additional file 10 Primers for CDS, GFP fusion and point mutated fragments amplification. Click here for file

Additional file 11 Primers used for 3′ RACE, 5′ RACE or 5′ RACE mapping of miRNA slicing sites confirmations. Click here for file

Additional file 12 **NCBI accession number of *AtAGO *genes.** Click here for file

Additional file 13 **Primers for PCR to detect expression of each *SlAGO *genes in collected tissues of tomato.** Click here for file
