# Supplementary material for: Molecular cloning and characterisation of SlAGO family in tomato
Source: BMC Plant Biol. 2013 Sep 8;13:126. doi: 10.1186/1471-2229-13-126 (PMC3847217; doi:10.1186/1471-2229-13-126)
Supplement: Additional file 1 — Localization and exon-intron mapped in chromosomes of SlAGO genes. [file 1471-2229-13-126-S1.doc]

| Gene name | Chromosome | Location | Exon number |
| --- | --- | --- | --- |
| SlAGO1A | 6 | 40969572-40960748 | 22 |
| SlAGO1B | 3 | 54075042-54084092 | 21 |
| SlAGO2A | 2 | 33782097-33788867 | 5 |
| SlAGO2B | 2 | 33791914-33795821 | 3 |
| SlAGO3 | 2 | 33797917-33801854 | 3 |
| SlAGO4A | 1 | 2976344-2984956 | 22 |
| SlAGO4B | 6 | 41722803-41731252 | 22 |
| SlAGO4D | 1 | 79534263-79540867 | 22 |
| SlAGO5 | 6 | 42661358-42667875 | 21 |
| SlAGO6 | 7 | 57105896-57093802 | 22 |
| SlAGO7 | 1 | 6612270-6616333 | 5 |
| SlAGO10 | 12 | 1214211-1199862 | 23 |
| SlAGO10A | 9 | 63895090-63888840 | 20 |
