# Supplementary material for: Molecular cloning and characterisation of SlAGO family in tomato
Source: BMC Plant Biol. 2013 Sep 8;13:126. doi: 10.1186/1471-2229-13-126 (PMC3847217; doi:10.1186/1471-2229-13-126)
Supplement: Additional file 2 — Basic informations of 13 cloned SlAGO genes. [file 1471-2229-13-126-S2.doc]

| Gene name | Predicted cDNA | ESTs | Length of cloned fragments | 3’RACE(3’UTR) | 5’RACE(5’UTR) |
| --- | --- | --- | --- | --- | --- |
| SlAGO1A | [Solyc06g072300.1.1](http://solgenomics.net/tools/blast/show_match_seq.pl?blast_db_id=142;id=Solyc06g072300.1.1;hilite_coords=1-3165) | [**SGN-U570489**](http://solgenomics.net/search/unigene.pl?unigene_id=SGN-U570489)**,** [**SGN-U588761**](http://solgenomics.net/search/unigene.pl?unigene_id=SGN-U588761)**,** [**SGN-U588762**](http://solgenomics.net/search/unigene.pl?unigene_id=SGN-U588762) | **3546bp** | **N(185bp)** | **Y(188bp)** |
| SlAGO1B | [Solyc03g098280.1.1](http://solgenomics.net/tools/blast/show_match_seq.pl?blast_db_id=142;id=Solyc03g098280.1.1;hilite_coords=110-2943,1-112) | [**SGN-U570490**](http://solgenomics.net/search/unigene.pl?unigene_id=SGN-U570490)**,**  [**SGN-U563432**](http://solgenomics.net/search/unigene.pl?unigene_id=SGN-U563432)**,** [**SGN-U566185**](http://solgenomics.net/search/unigene.pl?unigene_id=SGN-U566185)**,**  [**SGN-U595716**](http://solgenomics.net/search/unigene.pl?unigene_id=SGN-U595716) | **3729bp** | **N(90bp)** | **Y(183bp)** |
| SlAGO2A | [**Solyc02g069260.1.1**](http://solgenomics.net/tools/blast/show_match_seq.pl?blast_db_id=142;id=Solyc02g069260.1.1;hilite_coords=893-3129,328-917,39-269) | [**SGN-U567583**](http://solgenomics.net/search/unigene.pl?unigene_id=SGN-U567583) | **3557bp** | **N(326bp)** | **Y(50bp)** |
| SlAGO2B | [**Solyc02g069270.2.1**](http://solgenomics.net/tools/blast/show_match_seq.pl?blast_db_id=155;id=Solyc02g069270.2.1;hilite_coords=620-2934,1-621) | **NT** | **2995bp** | **N** | **N** |
| SlAGO3 | [Solyc02g069280.2.1](http://solgenomics.net/tools/blast/show_match_seq.pl?blast_db_id=155;id=Solyc02g069280.2.1;hilite_coords=1-3110) | **NT** | **3376bp** | **Y(83bp)** | **Y(270bp)** |
| SlAGO4A | [**Solyc01g008960.1.1**](http://solgenomics.net/tools/blast/show_match_seq.pl?blast_db_id=142;id=Solyc01g008960.1.1;hilite_coords=1-2730) | [**SGN-U577343**](http://solgenomics.net/search/unigene.pl?unigene_id=SGN-U577343)**,** | **3206bp** | **N(336)** | **Y(140)** |
| SlAGO4B | [**Solyc06g073540.1.1**](http://solgenomics.net/tools/blast/show_match_seq.pl?blast_db_id=142;id=Solyc06g073540.1.1;hilite_coords=1-2742) | [**SGN-U580584**](http://solgenomics.net/search/unigene.pl?unigene_id=SGN-U580584)**,** [**SGN-U576575**](http://solgenomics.net/search/unigene.pl?unigene_id=SGN-U576575)**,** [**GN-U579537**](http://solgenomics.net/search/unigene.pl?unigene_id=SGN-U579537) | **3120bp** | **N(270)** | **Y(104)** |
| SlAGO4D | [**Solyc01g096750.1.1**](http://solgenomics.net/tools/blast/show_match_seq.pl?blast_db_id=142;id=Solyc01g096750.1.1;hilite_coords=1-2646) | **NT** | **2691bp** | **N** | **Y(45)** |
| SlAGO5 | [**Solyc06g074730.1.1**](http://solgenomics.net/tools/blast/show_match_seq.pl?blast_db_id=142;id=Solyc06g074730.1.1;hilite_coords=1-3036) | [**SGN-U562884**](http://solgenomics.net/search/unigene.pl?unigene_id=SGN-U562884)**,** | **3246bp** | **N(140)** | **Y(69)** |
| SlAGO6 | [Solyc07g049500.1.1](http://solgenomics.net/tools/blast/show_match_seq.pl?blast_db_id=142;id=Solyc07g049500.1.1;hilite_coords=1-2712) | [**SGN-U568796**](http://solgenomics.net/search/unigene.pl?unigene_id=SGN-U568796)**,** [**SGN-U599050**](http://solgenomics.net/search/unigene.pl?unigene_id=SGN-U599050) | **2881bp** | **N** | **Y(169)** |
| SlAGO7 | [**Solyc01g010970.1.1**](http://solgenomics.net/tools/blast/show_match_seq.pl?blast_db_id=142;id=Solyc01g010970.1.1;hilite_coords=206-3003,87-145) | **NT** | **3238bp** | **Y(170)** | **Y(48)** |
| SlAGO10 | [**Solyc12g006790.1.1**](http://solgenomics.net/tools/blast/show_match_seq.pl?blast_db_id=142;id=Solyc12g006790.1.1;hilite_coords=1-2802) | [**SGN-U563343**](http://solgenomics.net/search/unigene.pl?unigene_id=SGN-U563343) | **3315bp** | **Y(157)** | **Y(391)** |
| SlAGO10A | [Solyc09g082830.1.1](http://solgenomics.net/tools/blast/show_match_seq.pl?blast_db_id=142;id=Solyc09g082830.1.1;hilite_coords=18-1741,1740-2949) | [**SGN-U604148**](http://solgenomics.net/search/unigene.pl?unigene_id=SGN-U604148)**,** | **3160bp** | **N(114)** | **Y(52)** |
