# Supplementary material for: Molecular cloning and characterisation of SlAGO family in tomato
Source: BMC Plant Biol. 2013 Sep 8;13:126. doi: 10.1186/1471-2229-13-126 (PMC3847217; doi:10.1186/1471-2229-13-126)
Supplement: Additonal file 3 — Information of SlAGO proteins and NCBI access numbers. [file 1471-2229-13-126-S3.doc]

| Gene name | ORF length | Polypeptide length | chromosome | Domains | Accession NO. |
| --- | --- | --- | --- | --- | --- |
| SlAGO1A | 3165 | 1054 | 6 | DUF1785，PAZ，Piwi | JX467704 |
| SlAGO1B | 3459 | 1152 | 3 | DUF1785，PAZ，Piwi | JX467705 |
| SlAGO2 | 3165 | 1054 | 2 | DUF1785，PAZ，Piwi | JX467706 |
| SlAGO2B | 2064 | 687 | 2 | DUF1785，PAZ，Piwi | JX467707 |
| SlAGO3 | 3000 | 999 | 2 | DUF1785，PAZ，Piwi | JX467708 |
| SlAGO4A | 2730 | 909 | 1 | DUF1785，PAZ，Piwi | JX467709 |
| SlAGO4B | 2743 | 913 | 6 | DUF1785，PAZ，Piwi | JX467710 |
| SlAGO4D | 2646 | 881 | 1 | DUF1785，PAZ，Piwi | JX467711 |
| SlAGO5 | 3036 | 1011 | 6 | DUF1785，PAZ，Piwi | JX467712 |
| SlAGO6 | 2712 | 903 | 7 | DUF1785，PAZ，Piwi | JX467713 |
| SlAGO7 | 3003 | 1000 | 1 | DUF1785，PAZ，Piwi | JX467714 |
| SlAGO10 | 2820 | 939 | 12 | DUF1785，PAZ，Piwi | JX467715 |
| SlAGO10A | 2880 | 959 | 9 | DUF1785，PAZ，Piwi | JX467716 |
