# Supplementary material for: Molecular cloning and characterisation of SlAGO family in tomato
Source: BMC Plant Biol. 2013 Sep 8;13:126. doi: 10.1186/1471-2229-13-126 (PMC3847217; doi:10.1186/1471-2229-13-126)
Supplement: Additional file 4 — G-Q content in N terminal sequences before domain of DUF1785. [file 1471-2229-13-126-S4.doc]

| Gene name | G content | Q content | Total(aa) |
| --- | --- | --- | --- |
| SlAGO1A | 15.2% | 9.5% | 335 |
| SlAGO1B | 17.9% | 14.9% | 436 |
| SlAGO2A | 16.0% | 9.6% | 374 |
| SlAGO3 | 9.0% | 7.7% | 323 |
| SlAGO5 | 12.8% | 6.1% | 312 |
