# Supplementary material for: Molecular cloning and characterisation of SlAGO family in tomato
Source: BMC Plant Biol. 2013 Sep 8;13:126. doi: 10.1186/1471-2229-13-126 (PMC3847217; doi:10.1186/1471-2229-13-126)
Supplement: Additional file 5 — Information of putative SlmiR403. [file 1471-2229-13-126-S5.doc]

| Gene name | Mature sequence | Genomic localization of pre-miRNA |
| --- | --- | --- |
| SlmiR403 | CUAGAUUCACGCACAAGCUCG | [SL2.40ch01](http://solgenomics.net/tools/blast/show_match_seq.pl?blast_db_id=148;id=SL2.40ch01;hilite_coords=70879767-70879836): 70879819  —70879839 |
