# Supplementary figures and images for: Molecular cloning and characterisation of SlAGO family in tomato
Source: BMC Plant Biol. 2013 Sep 8;13:126. doi: 10.1186/1471-2229-13-126 (PMC3847217; doi:10.1186/1471-2229-13-126)

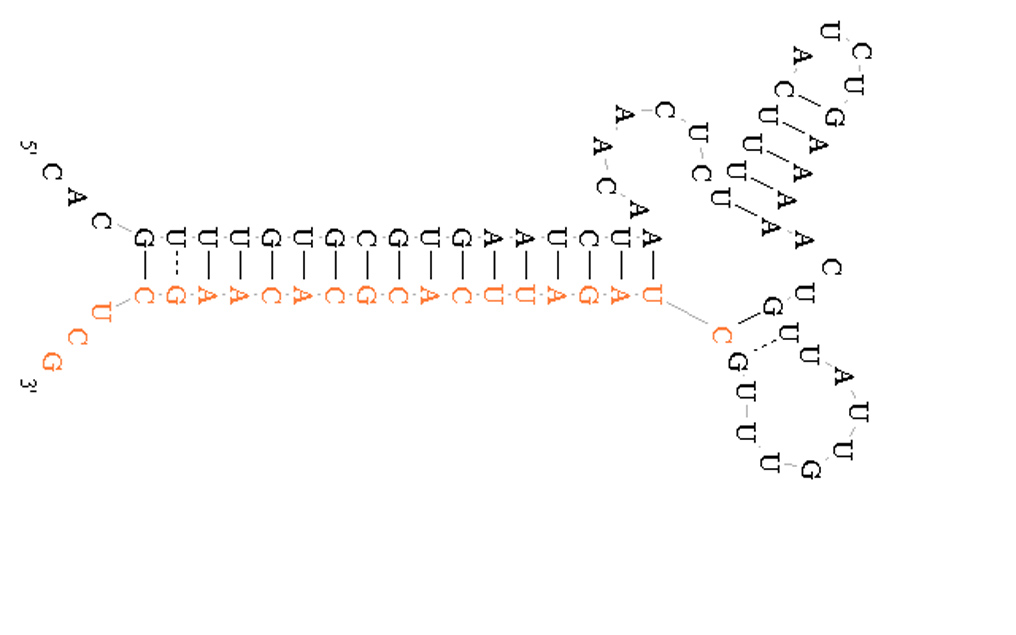

Supplement: Additional file 6 — Stem-loop structure of pre-SlmiR403. [file 1471-2229-13-126-S6.jpeg]

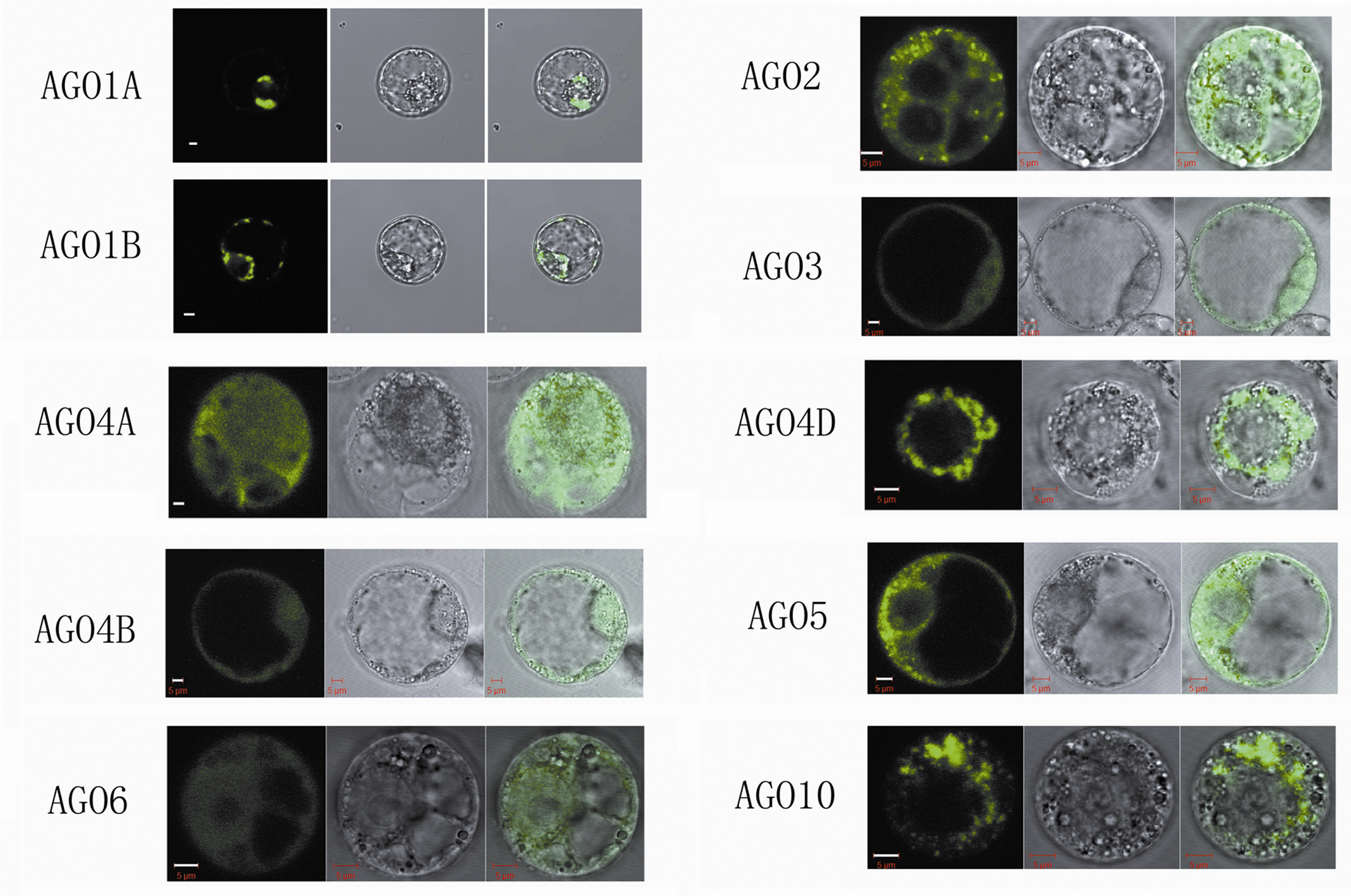

Supplement: Additional file 7 — Subcellular localization of SlAGO proteinss. [file 1471-2229-13-126-S7.jpeg]
