# Supplementary material for: Molecular cloning and characterisation of SlAGO family in tomato
Source: BMC Plant Biol. 2013 Sep 8;13:126. doi: 10.1186/1471-2229-13-126 (PMC3847217; doi:10.1186/1471-2229-13-126)
Supplement: Additional file 10 — Primers for CDS, GFP fusion and point mutated fragments amplification. [file 1471-2229-13-126-S10.doc]

| Primer | Sequence | Use |
| --- | --- | --- |
| AGO2B-F | ATGGACCGTGGAAACTACCAA | CDS amplification |
| AGO2B-R | GACAAAAAACATAATGTTCTGCAA | CDS amplification |
| AGO6-F | AGAGCAGTGGCGAAACAGAA | CDS amplification |
| AGO6-R | ACAGAAAAACATTGAGTCGCTGA | CDS amplification |
| AGO4C-F | ATGGCTGAACAAGAGTATGGT | CDS amplification |
| AGO4C-R | TCAACCAAAGACTATAACCCAG | CDS amplification |
| AGO4D-F | ATGGCCTCTTCAAAAGATGAA | CDS amplification |
| AGO4D-R | GCAGAAGAACATTGAACTGCGA | CDS amplification |
| AGO5-F | ATGTCGGAACGTGGACGA | CDS amplification |
| AGO5-R | CTGAATGTACGAAAAAGGAACAG | CDS amplification |
| AGO10A-F | GAAAGAAAGTTCAGAACAACACA | CDS amplification |
| AGO10A-R | ACAATAAAACATCACTCTCTTCAC | CDS amplification |
| AGO15-F | GAACAATCACGGAGGAGTACA | CDS amplification |
| AGO15-R | GCAGAAAAACATTGTATCGCG | CDS amplification |
| AGO1A-F | CTCTCCGTCTCATTTGCGTT | CDS amplification |
| AGO1A-R | CAGCATGTCCATATCATATAGCC | CDS amplification |
| AGO1B-F | TGTACGTTTTGGGTGCTGTGG | CDS amplification |
| AGO1B-R | GATCGCAAAACACAAAAACCAA | CDS amplification |
| AGO3-F | TCGCAAATGGACCGTGGAAA | CDS amplification |
| AGO3-R | GAACCGGACTGTCATCAAACGA | CDS amplification |
| AGO2A-R | GTAAAAGAAGCATGAAACCC | CDS amplification |
| AGO2A-F | GTGTTCACATTTGCTACTTTACTT | CDS amplification |
| AGO4A-F | GGTGCTTCATCTGCCTCTTT | CDS amplification |
| AGO4A-R | TCTTTCACATTACACCTGCTGG | CDS amplification |
| AGO4B-F | TACATTAGGGTTTCACCTCTTCACT | CDS amplification |
| AGO4B-R | ATAATTGATGCATAGTCACACCACC | CDS amplification |
| AGO10-F | TGTGAAGGCTAGGCAACAAAC | CDS amplification |
| AGO10-R | CATAAACTCAACATTCACCCTC | CDS amplification |
| AGO7-F | ACAACAAGTTATGGAACATACTGA | CDS amplification |
| AGO7-R | GCCATAACTTCAGCACTCAGCA | CDS amplification |
| AGO10A-REC-R | ACAATAAAACATCACTCTCTTCAC | GFP fusion |
| AGO4D-REC-R | GCAGAAGAACATTGAACTGCGA | GFP fusion |
| AGO6-REC-R | ACAGAAAAACATTGAGTCGCTGA | GFP fusion |
| AGO2B-REC-R | GACAAAAAACATAATGTTCTGCAA | GFP fusion |
| AGO2-REC-R | GACGAAAAACATTACGTTCTGCA | GFP fusion |
| AGO10-REC-R | GCAATAGAACATCACATTCTTCACCTT | GFP fusion |
| AGO5-REC-R | GCAGTAAAACATGACCTCCGA | GFP fusion |
| AGO3-REC-R | AACGAAAAACATTATGTCCTTCAAA | GFP fusion |
| AGO7-REC-R | GCAATAAAACATAAGCCTTTTAATG | GFP fusion |
| AGO4A-REC-R | GCAAAAGAACATAGAACTGGAAACT | GFP fusion |
| AGO1A-REC-R | ACAATAGAACATCACCCTTTTGAC | GFP fusion |
| AGO1B-REC-R | GCAATAGAACATGACCCTCTTCA | GFP fusion |
| AGO4B-REC-R | ACAGAAGAACATGGAGCTAGCAA | GFP fusion |
| AGO1AMUT-F | ACCAGTTCCAGAGCTCCATCAGGCAACTGAG | Piont mutation |
| AGO1AMUT-R2 | TTGCCTGATGGAGCTCTGGAACTGGTGGCCTAGAAG | Point mutation |
