# Supplementary material for: Molecular cloning and characterisation of SlAGO family in tomato
Source: BMC Plant Biol. 2013 Sep 8;13:126. doi: 10.1186/1471-2229-13-126 (PMC3847217; doi:10.1186/1471-2229-13-126)
Supplement: Additional file 11 — Primers used for 3′ RACE, 5′ RACE or 5′ RACE mapping of miRNA slicing sites confirmations. [file 1471-2229-13-126-S11.doc]

| Primer name | sequence | use |
| --- | --- | --- |
| AGO4D 5 GSP1 | CTCCAGGACAACATCAAACTCTAAC | 5' RACE |
| AGO4D 5 GSP2 | CCATCAGTGTTGTTCATCCCTAC | 5' RACE |
| AGO5 5 GSP1 | GCAGGGATGGTAGCAACAGGAGG | 5' RACE |
| AGO5 5 GSP2 | TCTGTTCAACCTCCCGAGTCATC | 5' RACE |
| AGO6 5 GSP1 | TCCAGTCTGTTGCGTGGTAGAGG | 5' RACE |
| AGO6 5 GSP2 | CCTCATCATTATAGTACGCTTGG | 5' RACE |
| AGO2B 5 GSP1 | GCCGTTGCCAAGCGTTACCACCA | 5' RACE |
| AGO2B 5 GSP2 | GTGGAGGCTGATGAAATGAACCC | 5' RACE |
| AGO10A 5 GSP1 | GTTCTTGATGACACTTCAGGGAT | 5' RACE |
| AGO10A 5 GSP2 | TTTGTCCCAAGTTGCCCATAACC | 5' RACE |
| AGO3 5 GSP1 | GCAGGGCCTTGTCTCTGTTCAT | 5' RACE |
| AGO3 5 GSP2 | GTGACCAGGAACACGATCAGA | 5' RACE |
| AGO2 5GSP1 | GGGCAGGTGCTCATTAGAGTAGAAACAACG | 5' RACE |
| AGO2B 5 GSP2 | CGTCAGACCAGTTCACAGCAAAACACCCAGTA | 5' RACE |
| AGO4B 5 GSP1 | ACAGAAGAACATGGAGCTAGCAA | 5' RACE |
| AGO4B 5 GSP2 | CATTAGGAGGGCTTCCATGTCTGC | 5' RACE |
| AGO4A 5 GSP1 | CCTTCCAACTCCCTTCCCATCAAC | 5' RACE |
| AGO4A 5 GSP2 | GCAGGAGTGAAATCTGGTGGAACA | 5' RACE |
| AGO7 5 GSP1 | GCACCTGATAGCATAACAGAATGGTCCTCC | 5' RACE |
| AGO7 5 GSP2 | GGTGGTTGCAGCAGCCCTGAC | 5' RACE |
| AGO10 5 GSP1 | TGTCCATATCCCGGCCTACGAG | 5' RACE |
| AGO10 5 GSP2 | CCTTTCCCTTTCCTCCTACCTCTTCTC | 5' RACE |
| AGO1A 5 GSP1 | CCGCACCATGATAGCAACTCAAA | 5' RACE |
| AGO1A 5 GSP2 | ATTTCCGCCCTTCTCCAACCAAC | 5' RACE |
| AGO1B 5 GSP1 | TGCCCTGCTGATAGTATTCA | 5' RACE |
| AGO1B 5 GSP2 | ATGTTGCTGGAGTGGTCCT | 5' RACE |
| AGO7 3 GSP1 | AGCAATTCGGCTGGCATGTTCG | 3' RACE |
| AGO7 3 GSP2 | CACGCTCTTCAACGAAAACATCCTACCAGG | 3' RACE |
| AGO10 3 GSP1 | GAGTGGGAACATCCTACCTGGTACTGT | 3' RACE |
| AGO10 3 GSP2 | TGCTCGTTGCACTCGTTCTGTTTC | 3' RACE |
| AGO3 3 GSP1 | CATCCTCTGAAGCTTCATCGTC | 3' RACE |
| AGO3 3 GSP2 | TGCACCATGATTTGAAGGACA | 3' RACE |
| AGO2-403GSP1 | GACACGTCTCAGAAATACATAATAC | 5' RACE for miR403 mapping |
| AGO1A-168 GSP1 | CTAAATGATGAAGATCAGCACGAGCA | 5' RACE for miR168 mapping |
| AGO1A-168 GSP2 | CCATCATCATCATCAAGAAGTGTGA | 5' RACE for miR168 mapping |
| AGO1B-168 GSP1 | CTCGGGAAGCGAATTTGATCACAACT | 5' RACE for miR168 mapping |
| AGO1B-168 GSP2 | CCATCCTCATCATCAGTAAGAGTGAT | 5' RACE for miR168 mapping |
