# Supplementary material for: Molecular cloning and characterisation of SlAGO family in tomato
Source: BMC Plant Biol. 2013 Sep 8;13:126. doi: 10.1186/1471-2229-13-126 (PMC3847217; doi:10.1186/1471-2229-13-126)
Supplement: Additional file 12 — NCBI accession number of AtAGO genes. [file 1471-2229-13-126-S12.doc]

| Gene name | Accession NO. |
| --- | --- |
| AtAGO1 | [NP_175274.1](http://www.ncbi.nlm.nih.gov/protein/15221177?report=genbank&log$=prottop&blast_rank=1&RID=2625K0XS01N) |
| AtAGO2 | NP_174413.2 |
| AtAGO3 | [NP_174414.1](http://www.ncbi.nlm.nih.gov/protein/15221662?report=genbank&log$=prottop&blast_rank=1&RID=262ES80701S) |
| AtAGO4 | NP_565633.1 |
| AtAGO5 | NM_179779.2 |
| AtAGO6 | NM_128854.3 |
| AtAGO7 | NP_177103.1 |
| AtAGO8 | NM_122111.2 |
| AtAGO9 | [CAD66636.1](http://www.ncbi.nlm.nih.gov/protein/28396616?report=genbank&log$=prottop&blast_rank=1&RID=262US0TG016) |
| AtAGO10 | NM_123748.2 |
