# Supplementary material for: Molecular cloning and characterisation of SlAGO family in tomato
Source: BMC Plant Biol. 2013 Sep 8;13:126. doi: 10.1186/1471-2229-13-126 (PMC3847217; doi:10.1186/1471-2229-13-126)
Supplement: Additional file 13 — Primers for PCR to detect expression of each SlAGO genes in collected tissues of tomato. [file 1471-2229-13-126-S13.doc]

| primer | sequence | Use |
| --- | --- | --- |
| RT AGO4B-F | CAGCACTTGGAGTACGAACC | PCR |
| RT AGO4B-R | TGTTCAGGAGAACAGAAGCG | PCR |
| RT AGO4A-F | GTTCCTCAGCTTCCAAAGCT | PCR |
| RT AGO4A-R | AGAGATAGCAAACCACAGAGCC | PCR |
| RT AGO1B-F | GGTGCAGGTTCTGCTGTTAG | PCR |
| RT AGO1B-R | CATGCGCCAAATGAAGTCT | PCR |
| RT AGO3-F | GCTTCATCGTCATCATCTTCA | PCR |
| RT AGO3-R | TGAAAAGGGCATCTCCAAAAT | PCR |
| RT AGO7-F | TTGAACGTTCCGATTTGTCT | PCR |
| RT AGO7-R | GCATGCAGTTGCCATAACTT | PCR |
| RT AGO10-F | GACTCACGTGACAACGGTTC | PCR |
| RT AGO10-R | TCAACATTCACCCTCTAGCAA | PCR |
| RT AGO2A-F | GGACGGATGTTTCAAGAGGT | PCR |
| RT AGO2A-R | GATCAACAAGGTGGCATCAG | PCR |
| RT AGO2B-F | GCGATGCACTAAACCTGTCT | PCR |
| RT AGO2B-R | CTGGGAATACATAATACGGGA | PCR |
| RT AGO6-F | CGACCTCTATTGTGGCTCCTG | PCR |
| RT AGO6-R | TGATCCTTGTTAGACGACCTTA | PCR |
| RT AGO4C-F | ATCAAGCCAGGATGGTCTGAGA | PCR |
| RT AGO4C-R | ACAGAAGAACATGGAGCTAGC | PCR |
| RT AGO4D-F | CTTCACAAGAATGTTCGCAGTT | PCR |
| RT AGO4D-R | GCTCCCAACAACTTCTATCCTAT | PCR |
| RT AGO5-F | TGCTAATGAAGGTGGTGAGG | PCR |
| RT AGO5-R | CTGAATGTACGAAAAAGGAACAG | PCR |
| RT AGO10A-F | ACGCTGGCATTCAGGTAACGA | PCR |
| RT AGO10A-R | GACAATTAACCTAGGATGATCAACA | PCR |
| RT AGO15-F | GTTATGCTCGTTTGGTTTCA | PCR |
| RT AGO15-R | TGAAAGTAAATGTAACCAATGCC | PCR |
| Ubi3-F | GCCGACTACAACATCCAGAAGG | PCR |
| Ubi3-R | TGCAACACAGCGAGCTTAACC | PCR |
